# Spontaneous Rectal Perforation With Transanal Small Bowel Evisceration: A Rare Complication of Chronic Rectal Prolapse

**DOI:** 10.7759/cureus.76904

**Published:** 2025-01-04

**Authors:** Harsh Harsh, Pankaj Kumar, Amit Karnik, Awanish Kumar

**Affiliations:** 1 General Surgery, King George's Medical University, Lucknow, IND

**Keywords:** emergency surgery, rectal prolapse, small bowel evisceration, solitary rectal ulcer, spontaneous rectal perforation

## Abstract

Small bowel evisceration through a rectal perforation is a rare but severe surgical emergency. It typically occurs following trauma or in elderly patients with a history of chronic constipation, rectal prolapse, prior abdominal surgeries, or multiple vaginal deliveries, which weaken the pelvic floor. While rectal and rectosigmoid perforations in patients with rectal prolapse are uncommon, the herniation of the small bowel through a perforation, resulting in evisceration per rectum, is an extremely rare presentation. Fewer than 100 cases of per rectal small bowel evisceration have been reported in the literature over the last 200 years, with the first case described in 1827. Among the published English literature, only 38 cases of nontraumatic rectal perforation with small bowel evisceration have been reported. Here, we present a rare case of spontaneous rectal perforation with transanal evisceration of the small bowel.

## Introduction

Transanal small bowel evisceration is a rare condition, typically occurring after trauma, but it can also be spontaneous. It requires prompt and appropriate treatment due to the high mortality rate if not managed adequately. Here, we present a rare case of spontaneous rectal perforation with transanal small bowel evisceration, along with a review of its pathophysiology and the appropriate surgical management.

## Case presentation

**Figure 1:**
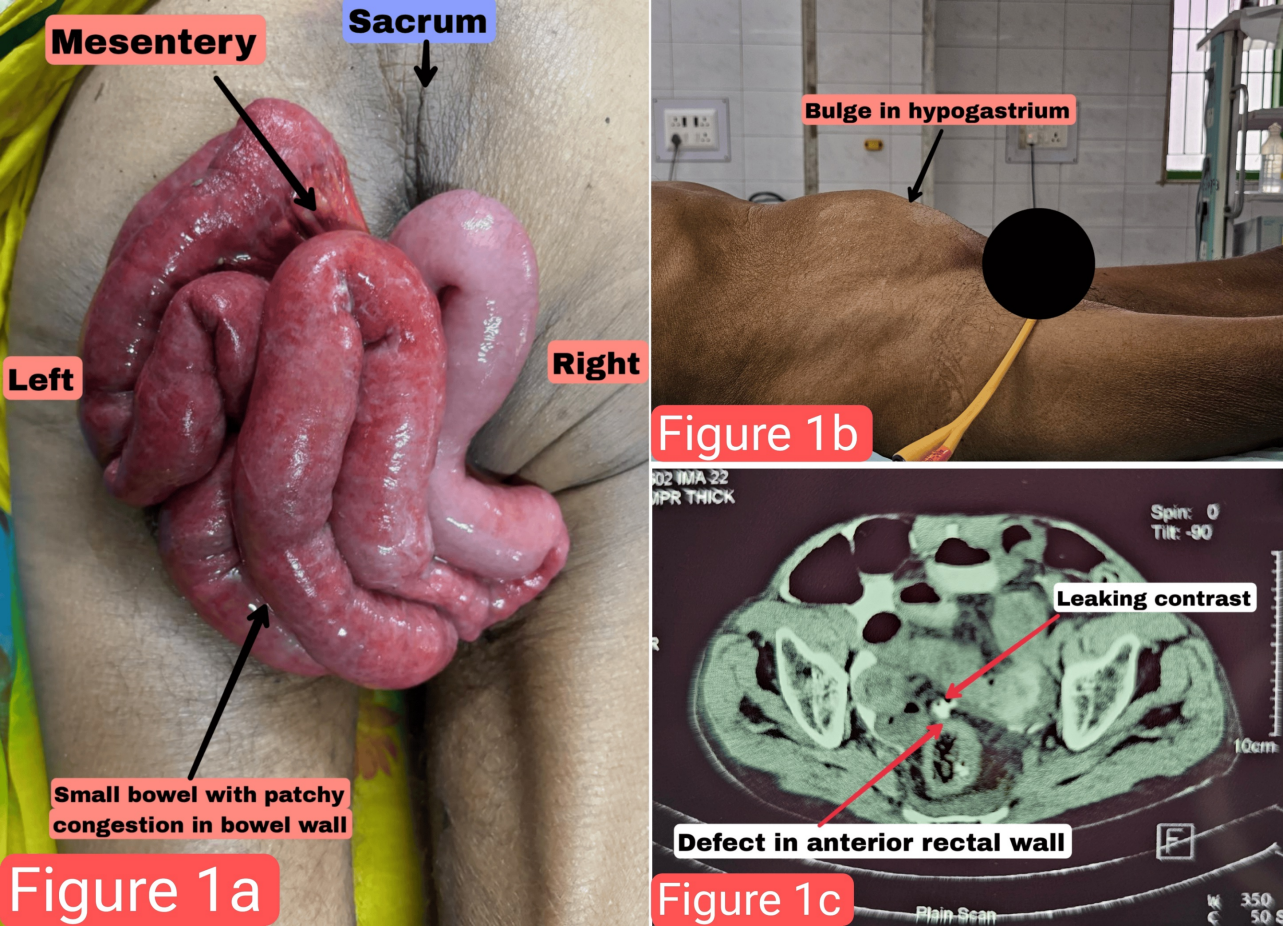
(a) Prolapsed small bowel. (b) Bulge in the hypogastrium. (c) Contrast-enhanced CT scan of the abdomen showing perforation on the anterior aspect of the upper rectal wall.

An elderly patient in his late 60s was brought to the emergency department with a complaint of sudden massive prolapse of the small bowel, which was irreducible manually and appeared abruptly while defecating in a squatting position (Figure 1a). He reported a longstanding history of chronic constipation with occasional lower abdominal pain and occasional rectal prolapse, which was spontaneously reduced. There was no history of per rectal penetration, rectal trauma, or blunt abdominal trauma.

On examination, the patient had stable vitals, with a blood pressure of 122/76 mmHg, a pulse rate of 102 per minute, a respiratory rate of 14 breaths per minute, and an oxygen saturation of 99% on room air. Abdominal examination showed a sunken appearance of the abdomen with mild tenderness in the hypogastrium (Figure 1b). Per rectal examination revealed an eviscerated small bowel, approximately three feet in length, along with its mesentery. No obvious ischemic changes were observed, though patchy congestion was present.

For initial management, the eviscerated bowel was washed with normal saline, covered with a glycerin-soaked gauze pad, and manually reduced back into the anal canal. Anal packing was performed to minimize the risk of ischemia. Digital rectal examination revealed no obvious rectal prolapse, and no rectal perforation was identified.

The patient remained vitally stable and was optimized for surgery. Imaging was planned, and a contrast-enhanced CT scan of the abdomen and pelvis with rectal and oral contrast was performed, revealing a perforation on the anterior aspect of the upper rectal wall (Figure 1c). Based on the clinical examination and imaging findings, a diagnosis of partial rectal prolapse with spontaneous rectal perforation and small bowel herniation was made.

**Figure 2:**
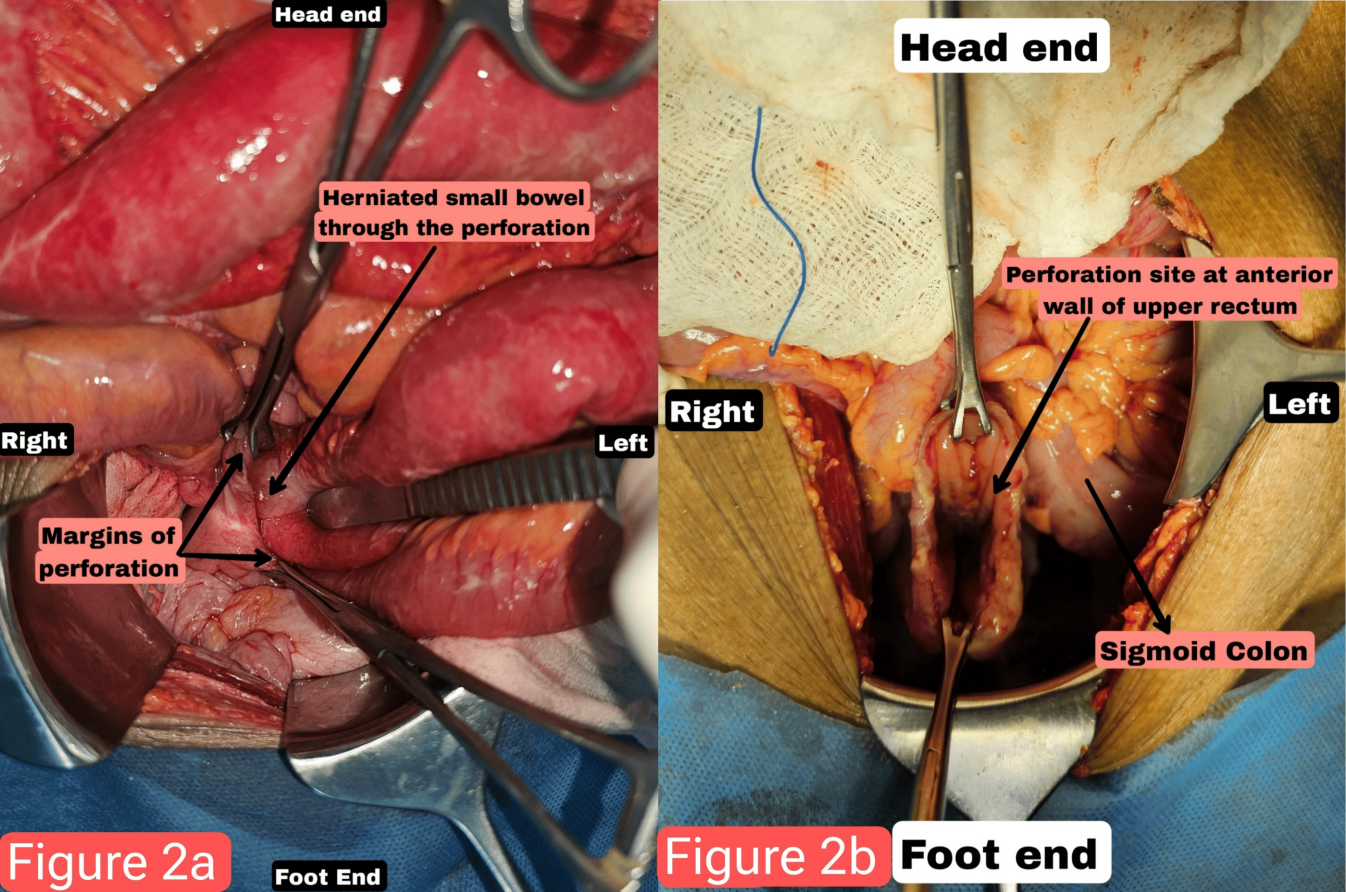
(a) Small bowel herniating through the rectosigmoid perforation. (b) Rectosigmoid perforation.

After optimization, the patient underwent definitive management in the form of laparotomy through a midline incision. The entire length of the small bowel was examined, and the terminal 2 ft of ileum was slightly congested but viable. The sigmoid colon was found to be redundant, measuring 70 cm, and a 5 cm longitudinal perforation was identified on the anterior wall at the rectosigmoid junction extending to the peritoneal fold (Figure 2a, 2b). The margins of the perforation were clear, with no visible growth; however, a biopsy of the margin was taken and sent for pathological examination to rule out malignancy, particularly in elderly patients. The perforation was repaired in two layers (inner continuous and outer interrupted) using polyglactin suture, and a diverting loop ileostomy was created in the terminal ileum, proximal to the ileocecal junction.

The postoperative period was uneventful, and the ileostomy began functioning on the first postoperative day, allowing for the initiation of oral intake. The patient was discharged on postoperative day 7 with a healthy surgical wound and instructions for follow-up care, pelvic floor exercises, and ileostomy reversal.

**Figure 3:**
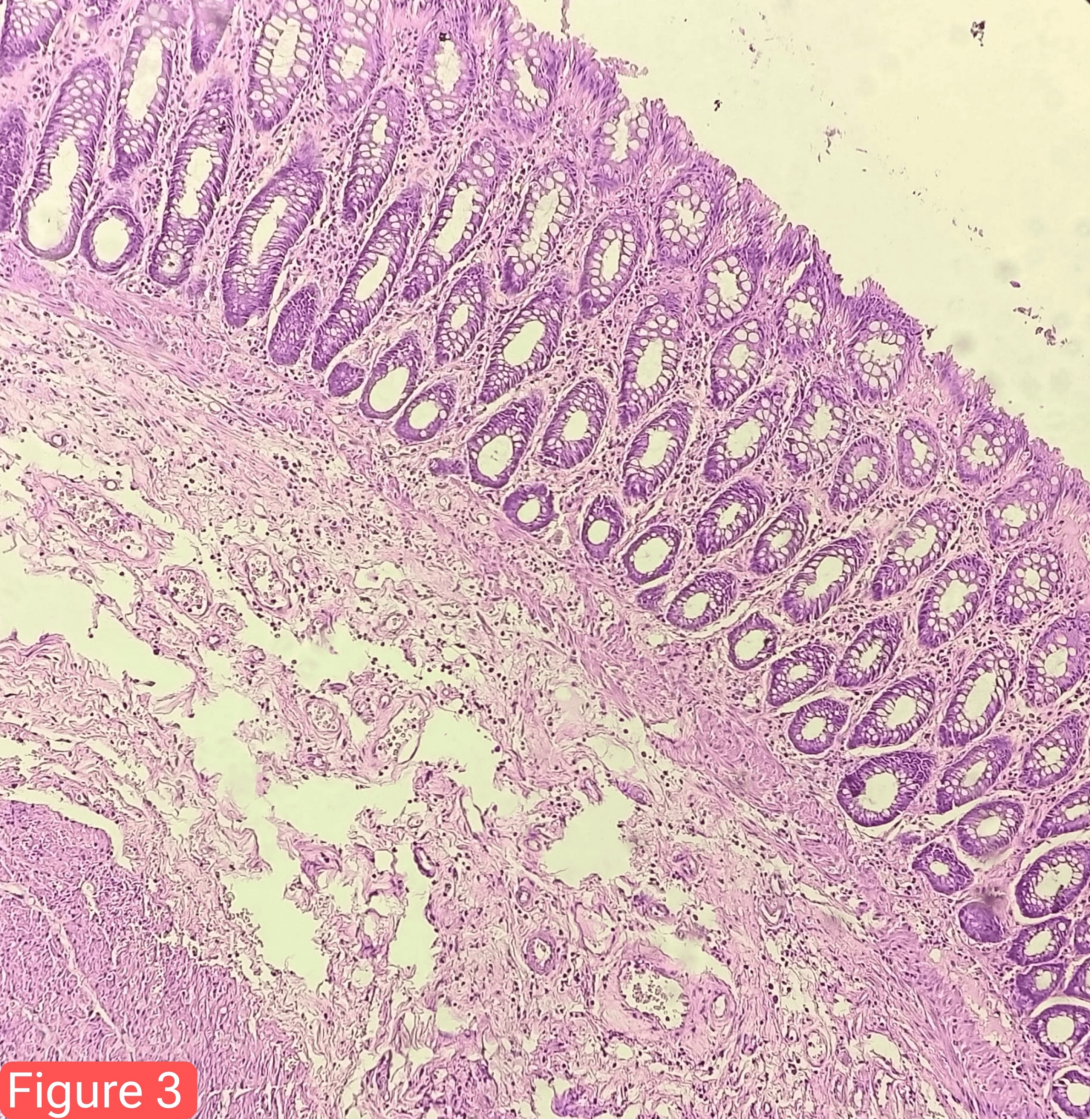
Colonic biopsy tissue showing focal ulceration in the colonic mucosal epithelial lining. The crypt architecture is unremarkable. The lamina propria shows chronic inflammatory infiltrate. The muscularis mucosa appears normal. Dense serositis is also noted. No evidence of granuloma or malignancy.

On follow-up after one month, the patient was nutritionally well, with a healthy surgical wound and a functional stoma. The histopathology report was nonmalignant and indicated chronic nonspecific inflammatory changes (Figure 3). A plan for a two-stage definitive surgery was discussed, involving resection rectopexy followed by ileostomy closure.

## Discussion

Small bowel evisceration per rectum through a rectosigmoid perforation is a rare surgical emergency with a mortality rate as high as 42.3% [1]. Fewer than 100 cases of per rectal small bowel evisceration have been reported in the literature since the first case described by Dr. B. C. Brodie in 1827 [2]. Most of these cases occurred following trauma, including rectal penetration, foreign body insertion, blunt abdominal injury, iatrogenic injuries, failed attempts at forceful digital reduction of chronic rectal prolapse, suction injuries in children, prolapse with chronic decubitus ulcers, and pressure necrosis [3-5]. Over 75% of these cases were associated with chronic rectal prolapse, often triggered by a sudden increase in intra-abdominal pressure [6,7]. In our case, the condition was linked to rectal perforation associated with chronic rectal prolapse.

Rectal prolapse is a type of sliding hernia, where a pouch of Douglas containing visceral organs prolapses through the anterior rectal wall into the rectum. Initially, it is a recto-rectal hernia, followed by recto-anal intussusception. Repeated episodes of prolapse and friction on the anterior rectal wall lead to progressive ischemia and thinning of the mucosal lining, resulting in solitary rectal ulcer syndrome [8]. A sudden increase in intra-abdominal pressure from activities like defecation, straining during micturition, vomiting, coughing, parturition, weightlifting, or blunt trauma can cause perforation and small bowel evisceration through the perforated site. In our case, chronic constipation and rectal prolapse contributed to the susceptibility to rectal perforation, which was precipitated by defecation in a squatting position.

There are no specific guidelines for managing this condition, but the approach generally depends on the patient's overall condition. Mortality is 100% if left untreated or if the bowel is simply repositioned, but it drops to 46% when the bowel is reduced and the tear repaired. The addition of a covering stoma further reduces mortality to 23% [9].

Initial management involves resuscitation with intravenous fluids, antibiotics, thorough washing of the small bowel with normal saline, reduction of bowel edema using glycerin-soaked gauze, gentle digital reduction of the bowel, and rectal packing. Prompt surgical intervention is necessary, depending on the viability of the herniated bowel, contamination, associated comorbidities, and the patient’s general condition [10].

For definitive management, a midline laparotomy is performed, and the small bowel and mesentery are gently reduced back into the abdomen and inspected for viability. Nonviable bowel should be resected. The rectal perforation is then primarily closed in one or two layers with polyglactin sutures, followed by a covering loop colostomy or ileostomy. In cases of extensive rectal pathology, such as trauma, rectal resection with end colostomy may be required [10]. In our case, we performed primary repair of the perforation with a covering loop ileostomy placed 1 ft proximal to the ileocecal junction, considering the patient’s advanced age, the emergency nature of the procedure, and the uncertainty regarding the cause of the perforation.

Treatment of the underlying cause, such as rectopexy or Thiersch wiring for rectal prolapse, should be considered. However, we deferred Thiersch wiring due to its high failure rate and opted for pelvic floor exercises, given the lesser degree of prolapse in our case, and we plan to pursue definitive management of the rectal prolapse following histopathological confirmation [10].

In high-risk patients who are unfit for surgery, alternative methods such as gentle digital repositioning with transanal repair of the perforation and a covering stoma can be attempted [11]. Laparoscopic repair of rectal perforation with covering stoma has also been reported [12].

In a literature review by Hajiev et al., a total of 38 cases of spontaneous rectal perforation were reported up to 2021. Their clinical outcomes, based on different management protocols, were compared [13]. Among these cases, 66% (n = 25) were associated with rectal prolapse, and four cases had a history of surgery for rectal prolapse. No significant history was found in 8% (n = 3) of patients. Constipation was present in 40% of cases (n = 15), and four cases had both constipation and rectal prolapse. Defecation was the precipitating event in 60% of cases (n = 23), while digital manipulation contributed in 10% (n = 4), and no precipitating event was identified in 26% (n = 10). The median age of the patients was 81 years, and most patients were elderly females. Only two cases involved constipation and rectal prolapse, with defecation as a precipitating factor, and our case represents the third in the published literature.

## Conclusions

Small bowel evisceration through rectal perforation is a rare but life-threatening complication of chronic constipation and rectal prolapse. Early recognition, along with prompt and appropriate surgical management, is crucial for achieving a favorable outcome.
